# Optimization of a synoviocyte-targeted biologic for inflammatory arthritis in combination or bispecific administration with TNF inhibitors

**DOI:** 10.1172/jci.insight.192984

**Published:** 2025-09-30

**Authors:** Sterling H. Ramsey, Zixuan Zhao, Megan C. Lee, Thales Hein da Rosa, Ava C. Schneider, Miriam Bollmann, Nour Dada, Katie E. Frizzi, May M. Han, Jaeyeon Kim, Martina Zoccheddu, Nigel A. Calcutt, Gary S. Firestein, James W. Bryson, Mattias N.D. Svensson, Eugenio Santelli, Stephanie M. Stanford, Nunzio Bottini

**Affiliations:** 1 Department of Medicine, Altman Clinical and Translational Research Institute, UCSD, La Jolla, California, USA.; 2 Kao Autoimmunity Institute and Division of Rheumatology, Cedars-Sinai Medical Center, Los Angeles, California, USA.; 3 Department of Rheumatology and Inflammation Research, Institute of Medicine, Sahlgrenska Academy, and; 4 SciLifeLab, University of Gothenburg, Gothenburg, Sweden.; 5 Department of Pathology, UCSD, La Jolla, California, USA.; 6 Bryson Biomed, Inc., Asheville, North Carolina, USA.

**Keywords:** Autoimmunity, Cell biology, Arthritis, Proteoglycans, Rheumatology

## Abstract

Rheumatoid arthritis (RA) is a common systemic autoimmune disorder. Fibroblast-like synoviocytes (FLS) have emerged as an attractive target for nonimmunosuppressive RA therapy, but there are no approved drugs targeting FLS. The receptor protein tyrosine phosphatase sigma (PTPRS) negatively regulates FLS migration and has been proposed as a target for FLS-directed RA therapy. Here we examined the impact of sequence variations on efficacy of an FLS-targeted biologic composed of Fc-fused PTPRS IgG-like domains Ig1 and Ig2 (Ig1&2-Fc). Engineering the linker and Fc tag improved effectiveness of human Ig1&2-Fc in assays of FLS migration and a mouse model of arthritis. Treatment of mice with Ig1&2-Fc over 4 months revealed no signs of toxicity or organ pathology. Finally, we show potential of Ig1&2-Fc coadministration in combination or as a bispecific fusion with a tumor necrosis factor-α inhibitor. Combination treatment of mouse tumor necrosis factor receptor 2 (mTnfr2) with Ig1&2-Fc resulted in increased efficacy in suppressing arthritis beyond single-agent treatment. When administered as a dual-action bispecific, Ig1&2 fused to mTnfr2 proved more efficacious at suppressing arthritis than mTnfr2 alone. This study illustrates the potential of Ig1&2-Fc as a combination or bispecific therapy with disease-modifying antirheumatic drugs to improve patient outcomes in RA.

## Abstract 2

Optimization of PTPRS-targeted biologic for RA that reduces FLS migration and arthritis in mice and enhances TNF inhibitor efficacy as a combination or bispecific therapy

## Graphical abstract

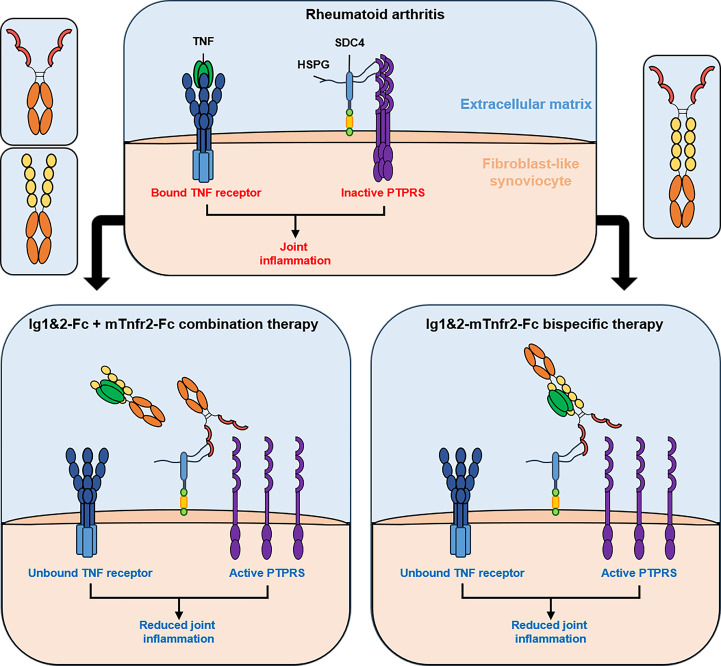

## Introduction

Rheumatoid arthritis (RA), characterized by chronic inflammation and degradation of joint cartilage and bone, is a common systemic autoimmune disorder (1). Development of immunosuppressant disease-modifying antirheumatic drugs (DMARDs) has substantially improved the treatment of RA (2). Among the most effective DMARDs are biologics blocking the action of the inflammatory cytokine tumor necrosis factor (TNF) by preventing its binding to the TNF receptors 1 and 2 (TNFR1 and TNFR2) (3). One such TNF inhibitor (TNFi) DMARD, etanercept, is an Fc fusion of the soluble region (p75) of human TNFR2. While TNFi and other DMARDs such as interleukin-6 (IL-6) inhibitors and Janus kinase inhibitors can be quite effective at treating RA, a large fraction of patients still does not reach a major clinical response with current therapies (4). As immunosuppressive agents, DMARDs are limited in their potential to be administered at higher doses or be combined due to unacceptable risks of infections and cancer. As such, there is a need for RA therapies that can be combined with current DMARDs to improve clinical response without further suppressing the immune system.

One potential approach to this problem has been to develop drugs targeting fibroblast-like synoviocytes (FLS). FLS are specialized nonimmune cells native to the synovium that contribute to joint health by providing structure and secreting synovial fluid and extracellular matrix (ECM). However, in the context of RA, FLS play a crucial role in RA pathogenesis through cartilage invasion, promotion of inflammation through secretion of cytokines such as IL-6, and degradation of the ECM through production of matrix metalloproteinases (5, 6). Although FLS have been proposed as a cell type of interest for developing nonimmunosuppressive therapy for RA, there currently are no drugs targeting FLS approved for clinical use.

We previously identified the transmembrane protein tyrosine phosphatase receptor sigma (PTPRS) as a therapeutic candidate for FLS-targeted RA treatment (6). PTPRS is expressed in FLS (6, 7) and the nervous system (8, 9), where its functionality is modulated by interaction between its N-terminal extracellular IgG-like domains 1 and 2 (Ig1&2) and proteoglycans (PGs) in the ECM (8–10). This mechanism of PTPRS regulation by ECM PGs has been termed the “PG switch” (8). PTPRS expression is nearly absent from the immune system, with the exception of plasmacytoid dendritic cells (11). We demonstrated that disruption of the interaction between PTPRS and the heparan sulfate PG syndecan-4 (SDC4) in FLS by treatment with recombinant Ig1&2 protein impaired FLS migration, invasiveness, and attachment to cartilage (6, 12). We further demonstrated that Ig1&2 suppresses arthritis in a PTPRS-specific manner in the K/BxN serum transfer-induced arthritis (STIA) model (6). Ig1&2 is also effective in 4 other mouse models of arthritis and operates through a nonimmunosuppressive mechanism (6, 12). Interestingly, combination treatment with Ig1&2 and a murine homolog of etanercept (mEta) synergistically improved therapeutic response in the collagen-induced arthritis (CIA) mouse model (12). Our previous findings demonstrate the potential of targeting FLS through a modulator of PTPRS that can also be combined with DMARDs to improve patient outcomes in RA.

Here, we performed engineering of the Ig1&2 protein (henceforth referred to as Ig1&2-Fc) to further its development as an RA therapeutic. We examined the impacts of Ig1&2-Fc protein modifications on FLS migration, arthritis severity in mice, and Ig1&2-Fc bioavailability in mice and explored Ig1&2-Fc as a combination therapy with mEta. We also report generation of a bispecific biologic fusion of Ig1&2 with mEta that combines the FLS-targeting action of Ig1&2-Fc with the immune-inhibitory properties of a TNFi.

## Results

### Effectiveness of Ig1&2-Fc sequence variations in assays of FLS migration.

**Figure 1:**
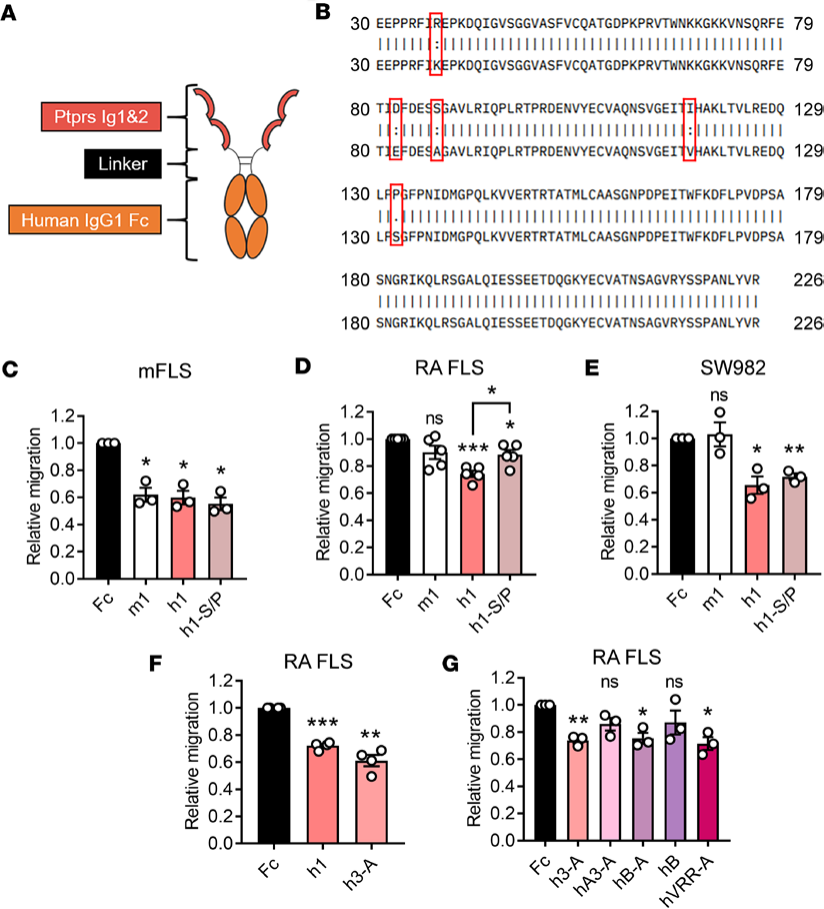
Inhibition of FLS migration by human Ig1&2-Fc proteins. (**A**) Scheme of Ig1&2-Fc. (**B**) Sequence alignment of murine (top) versus human (bottom) PTPRS Ig1&2 region. (**C**–**E**) Relative Transwell migration of mFLS (**C**; *n* = 3), RA FLS (**D**; *n* = 5), and SW982 cells (**E**; *n* = 3) in response to 10% FBS in the presence of Ig1&2-Fc or Fc control protein (100 nM). (**F**) Relative Transwell migration of RA FLS in response to 10% FBS in the presence of Ig1&2-Fc or Fc control protein (100 nM) (*n* = 4). (**G**) Relative Transwell migration of RA FLS in response to 10% FBS in the presence of Ig1&2-Fc or Fc control protein (100 nM) (*n* = 3). (**C**–**G**) Mean ± SEM relative to Fc control sample is shown. *P < 0.05, **P < 0.01, and ***P < 0.001; NS, nonsignificant; unpaired *t* test with Welch’s correction (**C**–**G**).

Our previously reported Ig1&2-Fc was composed of the Ig1 and Ig2 domains of murine Ptprs (amino acids 30–226 of NP_035348.2) linked to amino acids 221–447 (EU numbering) of human IgG1 Fc fragment (here called m1; Figure 1A) (6, 12). Sequence comparison of the mouse and human Ig1&2 region of PTPRS showed 5 amino acid variations. Four of these were conservative substitutions, while the fifth was a nonconservative S132P substitution (Figure 1B). We generated a human PTPRS Ig1&2-Fc (here called h1) and a human Ig1&2-Fc containing the murine P132 residue (here called h1-S/P; Table 1) and compared their effectiveness with m1 at inhibiting FLS migration. While all constructs were effective at inhibiting mouse FLS (mFLS) migration (Figure 1C), only h1 and h1-S/P were effective at inhibiting migration in RA FLS (Figure 1D) and SW982 cells (a human synovial sarcoma line; Figure 1E), with h1 showing greater efficacy to h1-S/P in RA FLS (Figure 1D). We therefore proceeded with human PTPRS Ig1&2-Fc.

Our previously reported m1 (6, 12) and our new h1 Ig1&2-Fc contain a 17 aa linker between Ig1&2 and Fc composed of a G_4_S repeat flanked by TS and 5 aa of the human IgG1 Fc hinge region (TSGGGGSGGGGSEPKSC). We examined a shortened linker with a single G_4_S motif (TSGGGGSEPKSC; here called h2) as well as a minimal linker (GT) with 2 mutations on the Fc as in the drug abatacept (C226S & C229S) (13) to reduce potential constraints (here called h3-A; Table 1). All variants were effective at inhibiting RA FLS migration; however, h3-A appeared to have a greater effect at higher concentrations (Figure 1F and Supplemental Figure 1; supplemental material available online with this article; https://doi.org/10.1172/jci.insight.192984DS1).

Human PTPRS contains 2 splice variants within the Ig1&2 region, resulting in incorporation of short “miniexons” A or B (14). Miniexon A encodes for inclusion of 9 aa (ETFESTPIR) in the Ig2 domain immediately after PTPRS residue S189. Miniexon B encodes for 4 aa (ELRE) immediately after PTPRS residue R226, following the Ig2 domain. We incorporated miniexon A into h3-A (here called hA3-A; Table 1) and found that it reduced effectiveness in the RA FLS migration assay (Figure 1G). Since miniexon B lies after the last Ig2 residue of PTPRS included in h3-A, we generated Ig1&2-Fc with miniexon B in replacement of the short linker, either with (hB-A) or without (hB) the Fc C/S mutations (Table 1). hB-A was similarly effective as h3-A at inhibiting RA FLS migration, while hB — which lacks the C/S mutations — was less effective (Figure 1G). We next generated another Ig1&2-Fc-C226/229S protein containing a 3 aa (VRR) natural extension of the canonical isoform of PTPRS in replacement of the short linker (hVRR-A; Table 1) and found it was similarly effective at inhibiting RA FLS migration as h3-A (Figure 1G). Taken together, results from these experiments suggest that proteins with short linkers between Ig1&2 and the Fc-C226/229S — as in h3-A — retain effectiveness in assays of FLS migration.

### Ig1&2-Fc does not display signs of toxicity in chronic treatment.

To further assess the potential of Ig1&2-Fc as a therapeutic for RA, we examined its toxicology after chronic administration. Previously, we only demonstrated its therapeutic effect in mouse models with acute treatment courses and did not characterize any potential side effects that arise from long-term exposure to Ig1&2-Fc. Due to potential limitations of administering a human biologic to mice for long-term toxicology studies, we generated a version of h3-A containing the mouse sequence of PTPRS (m3-A; Table 1). We first verified that this version of Ig1&2-Fc retained efficacy in mFLS. Both were effective at inhibiting mFLS migration, with m3-A performing slightly better than h3-A on the mouse cells (Supplemental Figure 1). Having verified its efficacy, we proceeded with m3-A as the Ig1&2-Fc for the toxicology studies described below.

**Figure 2:**
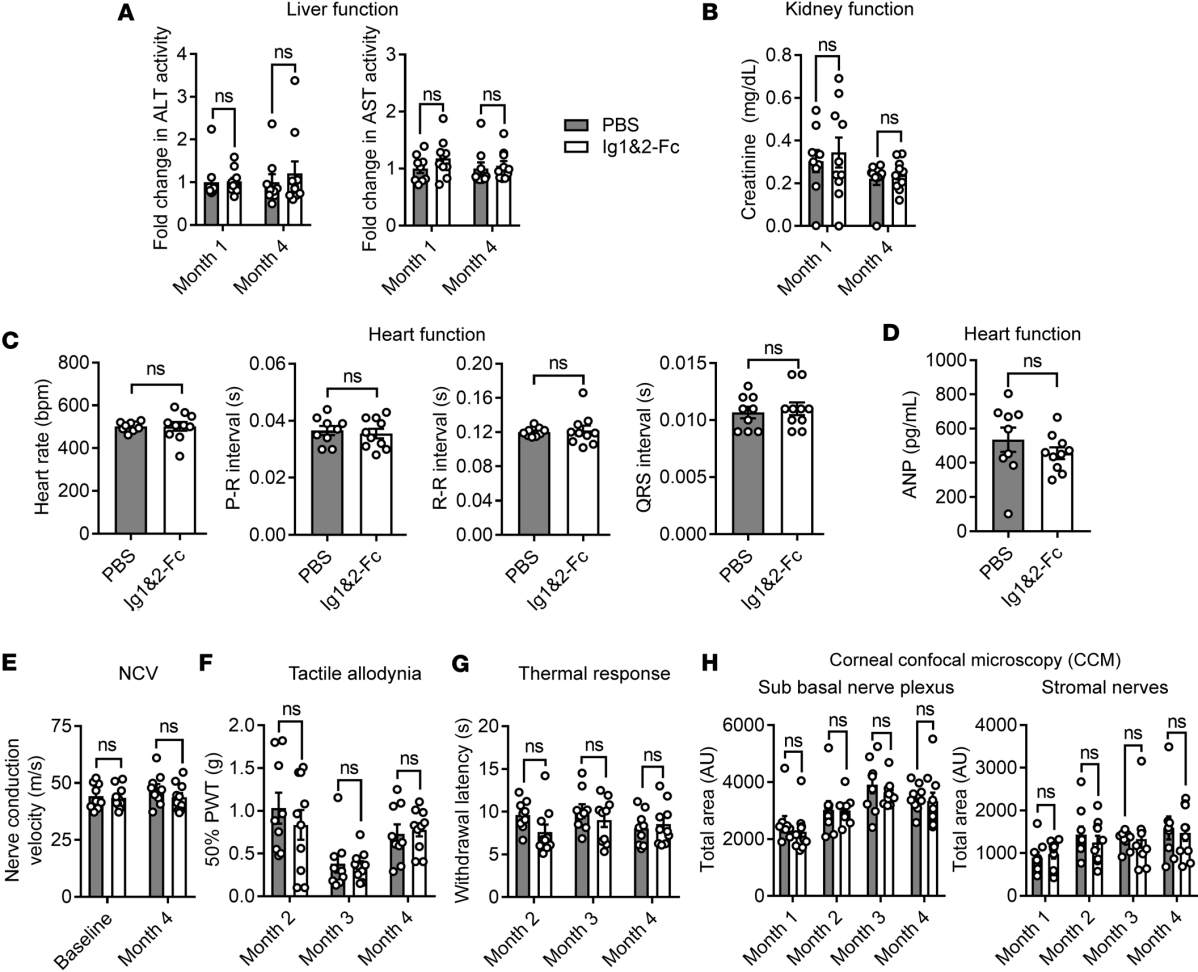
Four-month study shows no adverse effects of Ig1&2-Fc in mice. (**A**) Fold-change in serum alanine aminotransferase (ALT; left) and aspartate aminotransferase (AST; right) activity in mice treated with PBS or Ig1&2-Fc during months 1 and 4 of Ig1&2-Fc treatment. Mean ± SEM relative to PBS is shown. (**B**) Serum creatinine concentration in mice treated with PBS or Ig1&2-Fc during months 1 and 4 of treatment. (**C**) Electrocardiogram results of mice treated with PBS or Ig1&2-Fc after 4 months of treatment. (**D**) Serum atrial natriuretic peptide (ANP) in mice treated with PBS or Ig1&2-Fc after 4 months of treatment. (**E**) Nerve conduction velocity (NCV) of sciatic nerve in mice treated with PBS or Ig1&2-Fc before treatment (baseline) and after month 4 of treatment with Ig1&2-Fc. (**F**) Average force (in grams) of Von Frey filaments able to elicit a paw withdrawal response 50% of the time in mice treated with PBS or Ig1&2-Fc during months 2–4 of treatment (PWT, paw withdrawal threshold). (**G**) Paw withdrawal latency in response to heat of mice treated with PBS or Ig1&2-Fc during months 2–4 of treatment. (**H**) Total nerve area of sub basal nerve plexus (left) and stromal nerves (right) as observed by CCM during each month of treatment. (**A**–**H**) PBS: *n* = 9; Ig1&2-Fc: *n* = 10. Mean ± SEM is shown. NS, nonsignificant; 2-way ANOVA with Šídák’s correction (**A**, **B**, and **E**–**H**) or Mann-Whitney *U* test (**C** and **D**).

Eight- to 9-week-old mice were injected intraperitoneally every 2 days with PBS or 0.1 mg of Ig1&2-Fc for 4 months. Serum was collected every month to periodically assess for signs of loss of organ function. No elevation of circulating alanine aminotransferase (ALT) or aspartate aminotransferase (AST) was noticed in the mice treated with Ig1&2-Fc, indicating that liver function was not impaired (Figure 2A). Creatinine levels were similarly undisturbed, indicating no kidney toxicity (Figure 2B). Cardiac assessments were performed at the end of the study via electrocardiogram and echocardiogram, and no arrhythmias or signs of heart failure were detected in mice treated with Ig1&2-Fc (Figure 2C and Table 2). Circulating levels of atrial natriuretic peptide (ANP) were also similarly unaffected by Ig1&2-Fc administration (Figure 2D). Gross necropsy showed no correlation between organ pathology and Ig1&2-Fc treatment (Supplemental Table 1).

Because PTPRS is expressed in the nervous system (8, 9), we also assessed for peripheral neuropathy via nerve conduction, pain sensitivity studies, and assessment of corneal innervation. There was no difference in nerve conduction velocity (NCV) or paw withdrawal in response to external stimuli between mice that were treated with PBS or Ig1&2-Fc (Figure 2, E–G). Similarly, no alteration of corneal innervation was observed in mice treated with Ig1&2-Fc (Figure 2H). Taken together, these results suggest that chronic treatment with Ig1&2-Fc in mice up to 4 months does not result in evident toxicity or organ pathology as measured by this study. Having reaffirmed that Ig1&2-Fc is well tolerated in mice, we next turned to improve upon the efficacy of h3-A in vivo.

### Fc engineering improves Ig1&2-Fc efficacy against experimental arthritis in mice.

Next, we examined the effects of engineering the Ig1&2 IgG1-derived Fc fragment region of h3-A in efforts to improve its pharmacologic features. We generated 3 Fc-engineered h3-A proteins (Table 1). h3-A’ contained the neonatal Fc receptor (FcRn) affinity-enhancing M252Y/S254T/T256E (YTE) mutation reported to extend half-life (15) and the L234A/L235A (LALA) mutation to reduce potential binding to Fc-γ receptors (FcγRs) and consequent Fc-mediated cytotoxicity (16). h3-A-P/S contained a P238S mutation in the Fc also reported to prevent Fc-mediated cytotoxicity as in the LALA mutation (13). h3-A-P/S’ contained all 3 of these Fc-engineered mutations.

**Figure 3:**
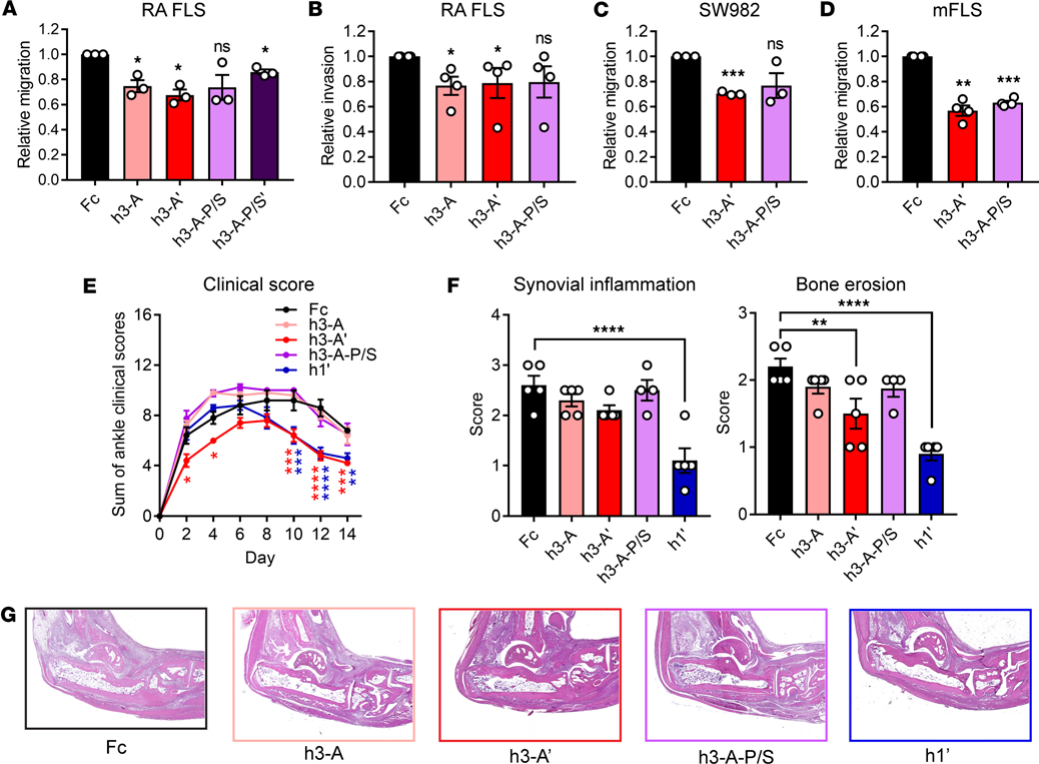
Additional Fc tag engineering improves Ig1&2-Fc effect in vivo. (**A**) Relative Transwell migration of RA FLS in response to 10% FBS in the presence of Ig1&2-Fc or Fc control protein (100 nM; *n* = 3). (**B**) Relative invasion through ECM of RA FLS in response to 10% FBS in the presence of Ig1&2-Fc or Fc control protein (100 nM; *n* = 4). (**C** and **D**) Relative Transwell migration of SW982 cells (**C**; *n* = 3) and mFLS (**D**; *n* = 4) in response to 10% FBS in the presence of Ig1&2-Fc or Fc control protein (100 nM). (**A**–**D**) Mean ± SEM relative to Fc control sample is shown. (**E**) Therapeutic treatment of BALB/c mice with K/BxN STIA by retro-orbital injection of 0.5 mg of Ig1&2-Fc or Fc control protein (h3-A-P/S: *n* = 4; others: *n* = 5) every 2 days for a total of 7 injections. (**F**) Histopathological evaluation of synovial inflammation (left) and bone erosion (right) from joints of mice in **E**. Average scores of both hind joints for each mouse are shown. (**G**) Representative H&E staining at 5× original magnification used for quantification in **F**. (**E** and **F**) Mean ± SEM is shown. *P < 0.05, **P < 0.01, ***P < 0.001, and ****P < 0.0001; NS, nonsignificant; unpaired *t* test with Welch’s correction (**A**, **C**, and **D**), Mann-Whitney *U* test (**B**), 2-way ANOVA with Dunnett’s correction (**E**), or 1-way ANOVA with Dunnett’s correction (**F**).

We first tested these proteins in assays of FLS migration to ensure that the efficacy of h3-A would not be impeded by the Fc mutations. h3-A’ was highly effective at inhibiting migration of RA FLS (Figure 3A) and had similar efficacy to h3-A at inhibiting RA FLS invasion through ECM (Figure 3B) and across scratch wound boundaries (Supplemental Figure 2). h3-A’ was also effective in SW982 cells (Figure 3C) and mFLS (Figure 3D). Together, these results suggest the YTE-LALA mutations do not interfere with the functionality of Ig1&2. Interestingly, inclusion of the P238S mutation in h3-A-P/S seemed to slightly hinder the inhibitory effect of Ig1&2 on human cell migration, although the protein retained consistent efficacy in the mouse FLS migration assay (Figure 3, A–D). Combining the 3 mutations in h3-A-P/S’ resulted in a deleterious effect compared with h3-A on the efficacy of the protein (Figure 3A). We therefore selected h3-A’ and h3-A-P/S for progression to in vivo experimentation.

We next tested the efficacy of h3-A’ and h3-A-P/S Ig1&2-Fc in the mouse STIA model in comparison with h3-A. Due to its similar ability to h3-A at inhibiting FLS migration at a lower concentration (Supplemental Figure 1), we also generated an Ig1&2-Fc incorporating the YTE-LALA mutations onto h1 (h1’; Table 1). As shown in Figure 3E, only h3-A’ and h1’ showed efficacy in mitigating arthritis when compared with Fc control protein. Histopathological evaluation of synovial inflammation and bone erosion in ankles from these mice further demonstrated the improved effect of the YTE-LALA mutations, as only h3-A’ and h1’ showed significantly reduced histopathological scores (Figure 3, F and G). Somewhat unexpectedly, h1’ had a superior effect compared with h3-A’. These results suggest that incorporation of YTE-LALA mutations improves the effect of human Ig1&2-Fc at suppressing inflammatory arthritis in mice.

### Bioavailability of Fc-engineered Ig1&2 proteins in mice.

**Figure 4:**
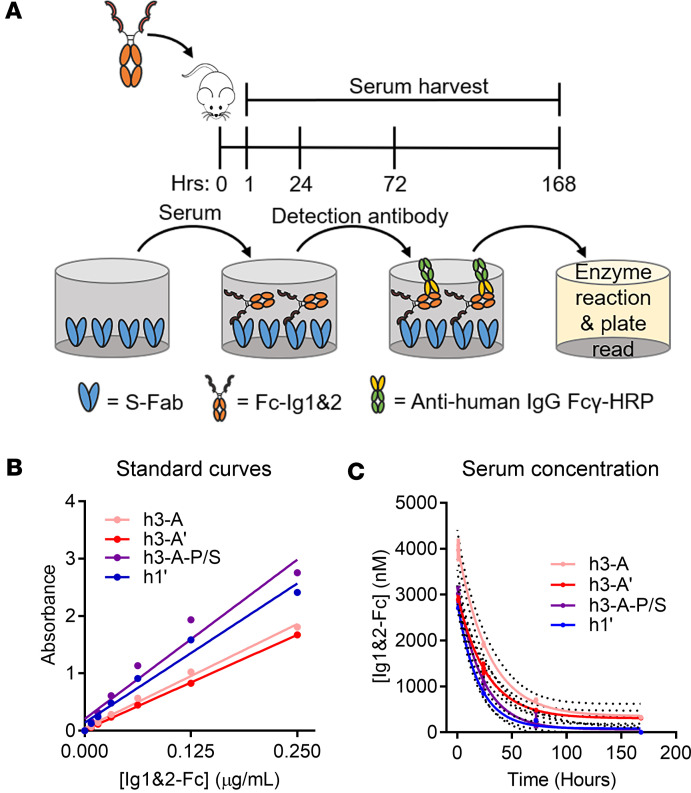
Half-life of Ig1&2-Fc proteins in vivo. (**A**) Schematic of the Ig1&2-Fc ELISA. Following retro-orbital injection with Ig1&2-Fc proteins, mouse serum was collected at the indicated time points. S-Fab was coated on high-binding, polystyrene, flat-bottom plates, followed by addition of diluted serum. HRP-conjugated anti-human IgG Fcγ was used as the detection antibody. (**B**) Standard curves of Ig1&2-Fc proteins were estimated by linear regression of the average of triplicate wells run at serial dilutions of 0.25–0.0078 μg/mL of Ig1&2-Fc. (**C**) In vivo half-life of Ig1&2-Fc protein. Concentration curves were estimated using nonlinear fit with 95% CI calculated with the 1-phase decay model (*n* = 3 for each time point).

Next, we sought to confirm whether the improved efficacy of Ig1&2-Fc in the mouse STIA model could be attributed to an improved half-life by examining the serum concentrations of these different Ig1&2-Fc proteins. To accomplish this, we generated an ELISA to capture and detect Ig1&2-Fc in mouse serum (Figure 4A). To capture Ig1&2, we generated a Fab fragment of an antibody clone recognizing the Ig1&2 region of PTPRS identified by phage display (here called S-Fab). To detect the Fc tag of the protein, we used an HRP-conjugated anti-human IgG Fc**γ** antibody (32935S; Cell Signaling Technology [CST]). We verified that S-Fab was able to detect Ig1&2-Fc in the presence of mouse serum by ELISA but did not nonspecifically bind to mouse serum matrix (Supplemental Figure 3).

Sera from mice that were administered a single 0.75 mg dose of Ig1&2-Fc protein via retro-orbital administration were collected at 1, 24, 72, and 168 hours postinjection. Serum levels of Ig1&2-Fc were analyzed by the ELISA as described above. For each different Ig1&2-Fc protein, a standard curve was run alongside the samples (Figure 4B) and used to calculate concentration at the indicated time points. h3-A had the highest concentration at each time point and showed a half-life of approximately 19.6 hours when the data were fit to a 1-phase decay nonlinear fit model (Figure 4C and Table 3). h3-A’ had a comparable half-life of approximately 18.4 hours. While inclusion of the P238S mutation in h3-A-P/S appeared to improve concentration levels at the earliest time point when compared with h3-A’, its levels dropped dramatically at the later time points, resulting in a deleterious effect on the half-life compared with h3-A. Incorporation of the YTE-LALA mutations did not lead to expected improvement of half-life in this model, although similar findings in mice have been reported (17). This was more evident in the half-life from mice administered h1’, which was even lower at approximately 13.3 hours. These results were interesting since both h3-A’ and h1’ were markedly more efficacious than h3-A at attenuating disease in the mouse STIA model, suggesting that incorporation of the YTE-LALA mutations nonetheless improves the therapeutic effect of Ig1&2-Fc in vivo.

### Combination treatment of Ig1&2-Fc with TNFi improves therapeutic response in mouse arthritis.

**Figure 5:**
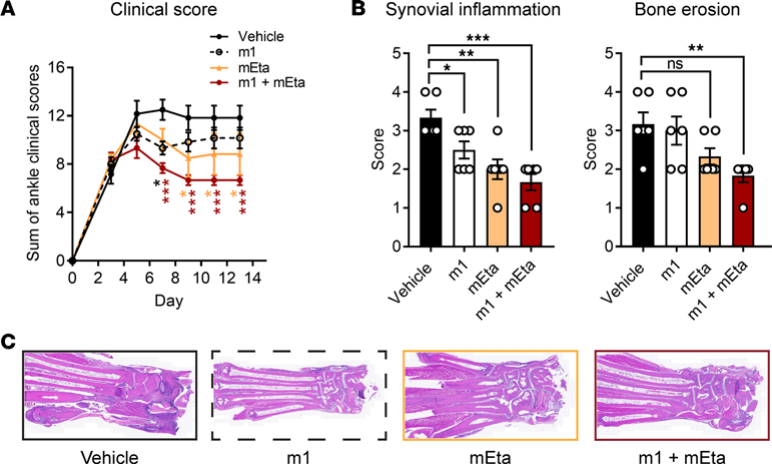
Combination treatment with Ig1&2-Fc and mTnfr2 improves therapeutic effect in mouse STIA model. (**A**) Therapeutic treatment of BALB/c mice with K/BxN STIA by intraperitoneal injection of 0.5 mg m1, 4 mg/kg mEta, combination of 0.5 mg m1 and 4 mg/kg mEta, or vehicle on days 0, 3, and every 2 days thereafter for a total of 6 injections (*n* = 6). (**B**) Histopathological evaluation of synovial inflammation (left) and bone erosion (right) from joints of mice in **A**. Scores from a single joint from each mouse are shown. (**C**) Representative H&E staining at 20× original magnification used for quantification in **B**. (**A** and **B**) Mean ± SEM is shown. *P < 0.05, **P < 0.01, ***P < 0.001; NS, nonsignificant; 2-way ANOVA with Dunnett’s correction (**A**) or 1-way ANOVA with Dunnett’s correction (**B**).

We previously reported that m1 Ig1&2-Fc shows additive efficacy with Fc-tagged p75 mTnfr2 (here called mEta) when the 2 biologics are coadministered together at suboptimal doses in the CIA model, a model of RA that is highly sensitive to TNFi (12, 18, 19). Here, we sought to assess Ig1&2-Fc efficacy in combination with optimal doses of mEta in the mouse STIA model, which is relatively insensitive to TNFi and models patients who achieve incomplete response with TNFi (20). We found that combination treatment of m1 and mEta suppressed arthritis in the mouse STIA model to a greater extent than single-agent treatment of either therapeutic (Figure 5A). This effect was further supported by histopathological evaluation of synovial inflammation and bone erosion in these mice (Figure 5, B and C). These results suggest that combining Ig1&2-Fc with etanercept or potentially other forms of TNFi can be used to improve therapeutic response in RA.

### Assessment of Ig1&2 fusion with TNFi as a bispecific therapy.

**Figure 6:**
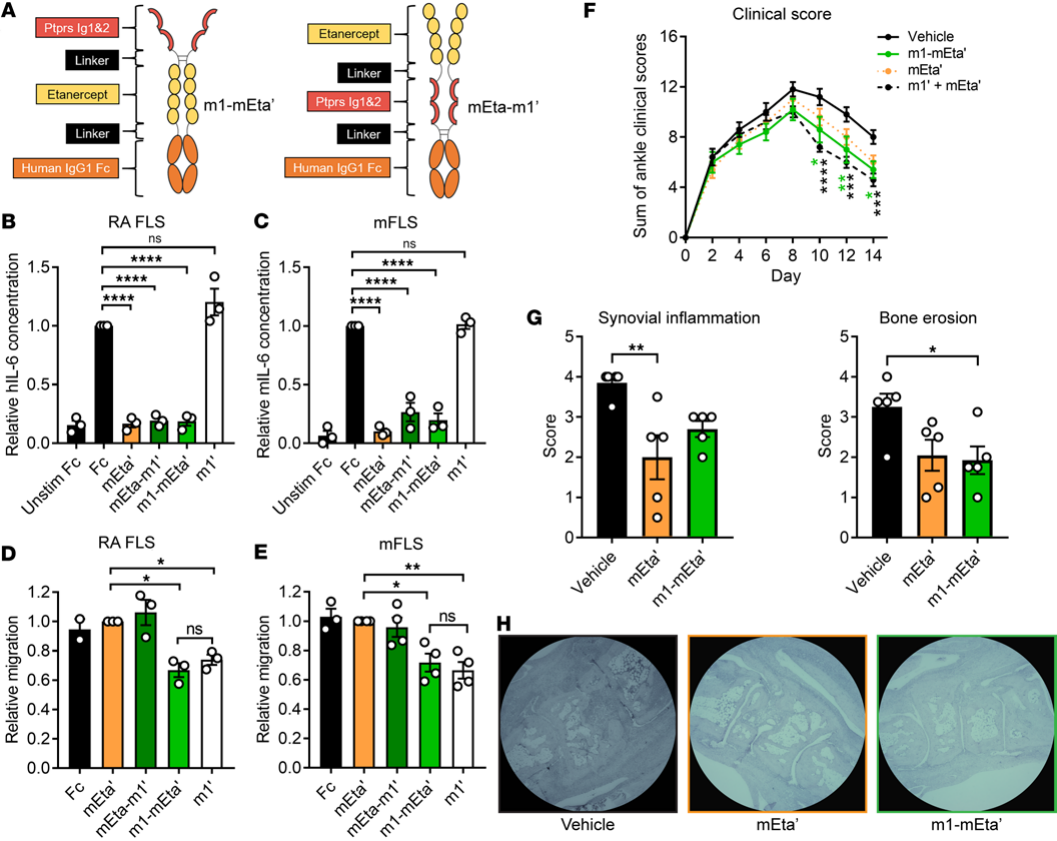
Bispecific fusion of Ig1&2 to mTnfr2 mimics therapeutic combination. (**A**) Scheme of Ig1&2 & TNFi fusion proteins. (**B** and **C**) Relative concentration of hIL-6 in RA FLS (**B**) or mIL-6 in mFLS (**C**) samples following 24-hour stimulation with 10 ng/mL TNF-α and the indicated protein (100 nM) (*n* = 3). Proteins were added 1 hour prior to stimulation. Mean ± SEM concentration relative to Fc control sample is shown. (**D** and **E**) Relative Transwell migration of RA FLS (Fc: *n* = 2; others: *n* = 3) (**D**) or mFLS (Fc: *n* = 3; others: *n* = 4) (**E**) in response to 10% FBS in the presence of the indicated protein (100 nM). Mean ± SEM relative to mEta’ sample is shown. (**F**) Therapeutic treatment of BALB/c mice with K/BxN STIA by intraperitoneal injection of 0.25 mg (10 mg/kg) m1-mEta’, 0.17 mg (6.8 mg/kg) mEta’, 0.17 mg (6.8 mg/kg) of both m1’ and mEta’, or vehicle every 2 days for a total of 7 injections (*n* = 5). (**G**) Histopathological evaluation of synovial inflammation (left) and bone erosion (right) of mice in **F**. Average scores of a single joint from each mouse by 2 scorers are shown. (**H**) Representative H&E images at 4× original magnification used for quantification in **G**. (**F** and **G**) Mean ± SEM is shown. *P < 0.05, **P < 0.01, ***P < 0.001, and ****P < 0.0001; NS, nonsignificant; 1-way ANOVA with Tukey’s correction (**B**–**E**), 2-way ANOVA with Dunnett’s correction (**F**), or 1-way ANOVA with Dunnett’s correction (**G**).

We next sought to examine the potential of fusing Ig1&2 with a TNFi DMARD to form a dual-action, bispecific therapeutic. We first incorporated the YTE-LALA mutations into our previously reported m1 Ig1&2-Fc (here called m1’) and mEta (here called mEta’). We then fused mEta to the N- or C-terminus of Ig1&2, resulting in mEta-m1’ and m1-mEta’, respectively (Figure 6A). These 4 proteins (m1’, mEta’, mEta-m1’, and m1-mEta’; Table 4) were tested in assays measuring IL-6 production in response to TNF and assays of FLS migration to assess retention of the functionality of each moiety. As expected, mEta’ abolished TNF-induced IL-6 production (Figure 6, B and C) but had no effect on FLS migration (Figure 6, D and E). On the other hand, m1’ significantly inhibited FLS migration but had no effect on IL-6 production (Figure 6, B–E). Both mEta-m1’ and m1-mEta’ blocked the response of cells to TNF, suggesting the addition of Ig1&2 at the N- or C-terminus is not deleterious to the TNF-blocking ability of mEta (Figure 6, B and C). Interestingly, insertion of mEta at the C-terminus in m1-mEta’ led to inhibition of FLS migration with the same effectiveness as m1’ — suggesting no interference from mEta on Ig1&2 function — while insertion of mEta at the Ig1&2 N-terminus in mEta-m1’ completely abolished its effect on FLS migration (Figure 6, D and E). Taken together, these data suggest addition of mEta to the Ig1&2 C-terminus results in a bispecific fusion protein with PTPRS and TNF targeting capabilities. We therefore selected m1-mEta’ for progression to in vivo experimentation.

Due to the increased molecular weight of m1-mEta’ (~74 kDa) compared with m1’ and mEta’ (~49 kDa and ~52 kDa, respectively), we opted to administer injections in molar equivalences of each therapeutic for in vivo experimentation. In mFLS, although m1-mEta’ was highly effective at inhibiting mIL-6 secretion, it appeared to have around 50% reduction in its inhibitory effect compared with mEta’ (Figure 6C); thus, we first sought to assess the effect of m1-mEta’ in reducing disease severity of the STIA model at twice the equimolar concentration to mEta’ in order to obtain similar functional activity of the mEta moieties in each protein. To obtain equivalent efficacies of the Ig1&2 moieties, equimolar doses of m1-mEta’ and m1’ were used. We found that m1-mEta’ and equimolar m1’ both significantly reduced arthritic severity, with our bispecific agent showing greater efficacy than m1’ alone (Supplemental Figure 4). At half the equimolar concentration to bispecific m1-mEta’, mEta’ did not significantly affect arthritis in the STIA model. These data suggest improved therapeutic efficacy of the bispecific m1-mEta’ compared with the action of each single component alone.

Having confirmed the effect of m1-mEta’ in vivo, we sought to evaluate the ability of bispecific m1-mEta’ to reduce disease severity in comparison with combination treatment with m1’ and mEta’. We thus compared the efficacy of m1-mEta’ versus single-agent mEta’ or coadministration of m1’ and mEta’ in the mouse STIA model at equimolar concentrations, finding that only m1-mEta’ and combination treatment of m1’ and mEta’ were able to significantly attenuate arthritis (Figure 6F). Histopathological evaluation of mice treated with mEta’ or m1-mEta’ showed that although equimolar mEta’ had a larger effect on synovial inflammation, m1-mEta’ showed stronger mitigation of bone erosion (Figure 6, G and H) — consistent with its FLS-targeting role. These results suggest the potential for further development of a bispecific fusion protein that mimics combination treatment of Ig1&2-Fc and TNFi within a single molecule to improve therapeutic response in RA.

## Discussion

While there is no doubt that DMARDs have drastically improved treatment of RA, there is still an unmet need to address patients who have an incomplete response to current therapy. FLS have emerged as promising cells of interest for developing new RA therapies because of the potential for regulating key mediators of RA disease progression in a nonimmunosuppressive manner (5, 6). We detail optimization and development of an FLS-targeted biologic, Ig1&2-Fc, a decoy protein consisting of the 2 N-terminal Ig-like domains of the receptor phosphatase PTPRS. Ig1&2-Fc competitively disrupts the binding of PTPRS to its negative regulator SDC4 on the surface of FLS, leading to activation of the inhibitory function of PTPRS and substantially reducing invasiveness of these cells (6, 12).

We show developments in the human Ig1&2-Fc protein that result in improved efficacy in RA FLS migration assays and the mouse STIA model. We also show that therapeutic response can be improved through coadministration of Ig1&2-Fc and a TNFi. Fusion of these 2 biologics results in a single molecule that retains the effective properties of Ig1&2-Fc and the TNFi in assays of FLS migration and TNF-induced IL-6 secretion, respectively, and leads to similar attenuation of inflammatory arthritis in mice to combination therapy.

To better develop recombinant PTPRS Ig1&2 as a therapeutic for RA, we sought to augment its design to more closely resemble human PTPRS. In doing so, we found that while species-specific differences in the Ig1&2 domain resulted in similar efficacy on inhibiting mouse FLS migration, there was a marked improvement on inhibiting migration in RA FLS and SW982 cells with the fully human version of Ig1&2. Introduction of a minimal linker and C226/229S mutations in the Fc tag appeared to slightly improve the efficacy of Ig1&2-Fc. This effect was consistent across multiple proteins with varying sequences in the Ig1&2 region, suggesting that proteins similar to h3-A are comparably effective at inhibiting FLS migration.

We show that chronic administration of Ig1&2-Fc did not cause any signs of organ toxicity in mice. Peripheral nerve function was similarly unaffected. As measured by the tests administered for our study, there do not appear to be any obviously harmful side effects from repeated long-term injections of Ig1&2-Fc. Additional tests in other nonhuman models with our improved and bispecific agents will be necessary to determine if there are any latent adverse side effects that were not observed here.

Many current Fc-containing biologics are reported to include mutations in the Fc region, such as M252Y/S254T/T256E (YTE) to improve drug availability and L234A/L235A (LALA) to reduce off-target cytotoxic effects (15, 16). The P238S Fc mutation has also been reported to prevent Fc-mediated cytotoxicity (13). To examine whether the in vivo efficacy of Ig1&2-Fc could be bolstered by these mutations, we first sought to verify that they would not interfere with the effect of Ig1&2-Fc on inhibiting FLS migration. We observed that addition of the YTE-LALA mutations (h3-A’) led to the most consistent and comparable efficacy to h3-A and was an obvious choice for in vivo progression. Inclusion of the P238S Fc variation (h3-A-P/S) resulted in slight interference of Ig1&2-Fc efficacy at inhibiting migration in the RA FLS and SW982 cells but still showed comparable efficacy to h3-A’ at inhibiting migration of mouse FLS. For this reason, h3-A-P/S was still considered for progression to mouse experiments. Inclusion of YTE-LALA with P238S (h3-A-P/S’) resulted in an undesirable worsening of the effect of Ig1&2-Fc and was not considered for further testing.

Following our cell-based Ig1&2-Fc optimization efforts, we selected proteins for in vivo administration in the mouse STIA model. h3-A, h3-A’, and h3-A-P/S were selected based on their performance in the FLS migration assays. Since the h1 protein showed comparable efficacy to h3-A in the RA FLS migration assays at 50 nM, we decided to also include a version of this protein with inclusion of the YTE-LALA mutations (h1’). Interestingly, the ability of human Ig1&2-Fc to suppress arthritis was dramatically improved by inclusion of the YTE-LALA mutations. We observed that administration of either h3-A’ or h1’ led to substantial reduction in arthritis in this model. Surprisingly, the 2 proteins lacking YTE-LALA, h3-A and h3-A-P/S, had no noticeable effect. Although unexpected, these findings suggest the human Ig1&2 sequence is less effective than the mouse version when used in the mouse model.

We next examined the effects of the YTE-LALA mutations on Ig1&2-Fc bioavailability in mice. Unexpectedly, h3-A’ showed a slightly reduced calculated half-life to h3-A (which showed no therapeutic effect in the mouse STIA model), and h1’ had the shortest calculated half-life of all measured proteins. This was surprising given that h3-A’ and h1’ showed substantially more attenuation of arthritis. These results could be attributed to previous findings that mutations which enhance IgG1 affinity to mouse FcRn at physiological pH reduce serum availability in mice when compared with wild-type IgG1 (17). It has also been reported that murine FcRn exhibits greater binding than human FcRn to human IgG1 (21), and some studies that measure the efficacy of the YTE and other IgG1 mutations on half-life opt to use human FcRn-transgenic mice (22–24). Therefore, we have reason to believe that incorporation of the YTE-LALA mutations, which nonetheless impart a positive impact on the therapeutic effect of Ig1&2-Fc, may affect the quantification of the protein concentrations in the serum because of limitations of the model species utilized in our study. Additional experimentation in further nonhuman models will help to more accurately assess the pharmacology of Ig1&2-Fc.

Considering the collective results of our efforts to further improve Ig1&2-Fc, it is interesting to consider the discrepancies between the observable effects of each iteration. Perhaps most notably, we noticed an enhanced effect of migratory inhibition in vitro with our preliminary testing of h3-A in comparison with h1, only to discover h3-A fails to adequately reduce severity of experimental arthritis in mice. Inclusion of the YTE-LALA mutations seemed to overcome this failure as in h3-A’, but, rather surprisingly, h1’ had the most profound effect at attenuating arthritis. It seems that incorporation of the longer linker in h1’ might afford our Ig1&2-Fc additional flexibility to retain proper biological function within the complex environment of a living system. Conversely, perhaps the more rigid structure of the minimal GT linker in h3-A and h3-A’ is better suited to the comparatively simplified conditions of in vitro experimentation. Further study is necessary to draw conclusions about the relative efficacy of Ig1&2-Fc between in vivo and in vitro administration, as well as between model species. However, from the data we present, it seemed appropriate to return to our previously reported m1 for preliminary development of combination treatment with DMARDs in models of mouse arthritis.

We previously reported that TNF downregulates PTPRS in RA FLS through a GSK3β-dependent pathway and that combination treatment of suboptimal doses of m1 with TNFi enhances the therapeutic response of each agent in the mouse CIA model (12). As the CIA model is exquisitely responsive to TNFi, it is not well suited to model additive effects of treatments that are meant to increase control of disease in patients with incomplete response to TNFi (18, 19). The STIA model, while modulated in part by TNF, is mostly IL-1 driven, and full-dose TNFi treatment may cause only a moderate decrease in disease activity (20). Here we assessed a combination of TNFi and our biologic in the STIA model, finding a similar additive effect when these biologics were administered together. This provides further support for the potential of an FLS-targeting therapy to be used in concert with DMARDs to enhance control of disease in partial responders.

Based on these results, we asked whether Ig1&2 could be fused to a biologic DMARD to allow for administration of both entities as a single molecule. Upon fusion, the TNFi retained its functional effect whether positioned on the N- or C-terminus of the molecule. However, the functional effect of Ig1&2 was only conserved when in the N-terminal position of the molecule (as in m1-mEta’). These results are consistent with our previous findings that Fc incorporation onto the Ig1&2 protein C-terminus does not have a deleterious effect on Ig1&2-Fc function in cell-based assays or in vivo (12).

Having confirmed our bispecific molecule was able to retain the function of its component biologics, we next sought to compare them in the STIA model. We saw a similar improved effect of the bispecific m1-mEta’ molecule at attenuating arthritis compared with combination administration of m1’ and mEta’. Future efforts will need to focus on validating the efficacy of a human bispecific Ig1&2-Fc, as well as optimizing the relative affinities of the Ig1&2 and TNFi moieties to potentially improve long-term therapeutic effect without the risk of overadministering immunosuppressive DMARDs.

In conclusion, we report an FLS-targeted biologic optimized for RA therapy and development of a proof-of-principle bispecific biologic that is similarly efficacious against FLS and inhibits TNF as a dual strategy for combating RA. We provide evidence for the efficacy of FLS-targeted treatment with Ig1&2-Fc as a monotherapy, in combination with a TNFi DMARD homolog, and as a single bispecific agent that retains both therapeutic characteristics of its single-action constituent parts. As such, we hope to provide further support for development of therapeutic strategies targeting FLS in RA.

## Methods

### Sex as a biological variable

Toxicology studies were performed in both male and female mice. Since RA disproportionately affects females, arthritis and bioavailability experiments were predominantly performed using female mice.

### Proteins and other reagents

#### Production of Ig1&2-Fc proteins.

Numbering of PTPRS sequences refers to accession NP_035348.2 for murine Ptprs or NP_570923.2 for human PTPRS. Numbering for human IgG1 Fc sequences refers to the EU numbering scheme (25). Preparation of Ig1&2-Fc proteins was contracted to Viva Biotech or LakePharma. Proteins were expressed in Chinese hamster ovary (CHO) cells and purified using a Protein A and subsequent HiTrap Heparin column (Cytiva) in 20 mM Tris with 150 mM NaCl, pH 7.2. All proteins were analyzed for purity using high-performance liquid chromatography (HPLC) and reducing and nonreducing SDS-PAGE and confirmed free of endotoxin (<1 EU/mg).

#### Production of anti-PTPRS Fab fragment (S-Fab).

S-Fab (the Fab of an antibody clone identified by phage display against the Ig1&2 region of PTPRS; Creative Biolabs) was produced by Viva Biotech. S-Fab was expressed in CHO cells and purified against a Protein L resin column in PBS, pH 7.4. S-Fab was analyzed for purity using HPLC and reducing and nonreducing SDS-PAGE and confirmed free of endotoxin (<1 EU/mg).

#### Other reagents.

Fc control protein was purchased from Invitrogen (A42561) for in vitro assays and from BioXCell (BE0096) for in vivo administration. Unless specified, other reagents and chemicals were purchased from Sigma-Aldrich.

### Cell line preparation and culture

Human RA FLS were obtained from the UCSD Clinical and Translational Research Institute Biorepository. Each line was previously obtained from discarded synovial tissue from patients with RA undergoing synovectomy, as approved by the UCSD IRB under protocol 140175. The diagnosis of RA conformed to the American College of Rheumatology 1987 revised criteria (26).

To generate mouse FLS, elbow, knee, and ankle joints were isolated from C57BL/6 mice. Tissues were minced and digested in collagenase IV (0.5 mg/mL) in RPMI 1640 (10-040-CV; Thermo Fisher Scientific) for 2 hours at 37°C with gentle agitation and cultured in complete DMEM (described below).

FLS and SW982 cells (acquired from the American Type Culture Collection; HTB-93) were cultured in DMEM with 4.5 g/L glucose and l-glutamine, without sodium pyruvate (DMEM; 10-017-CV; Corning), supplemented with 10% FBS (S11550H; Bio-Techne), gentamicin (15710072; Thermo Fisher Scientific), penicillin (100 U/mL), and streptomycin (100 μg/mL) (30-002-CI; Corning), at 37°C in a humidified atmosphere containing 5% CO_2_. Media were refreshed every 2–3 days. FLS were used between passages 4 and 9, and SW982 cells were kept in culture no longer than 4–5 weeks. Cells were synchronized in DMEM supplemented with 0.1% FBS overnight before experiments.

### Migration and invasion assays

#### Migration assays.

Migrations were performed similarly as described (12). Confluent cells were serum-starved overnight in DMEM containing 0.1% FBS, harvested by trypsin digestion, and seeded at 1.5 × 10^4^ cells in 100 μL of serum-free DMEM containing 0.5% bovine serum albumin (BSA; A30075-100.0; RPI Corp.) in the upper chamber of a 6.5 mm–diameter Transwell polycarbonate culture insert with a pore size of 8 μm (07-200-150; Corning). Inserts were placed in 24-well plates with 600 μL of DMEM containing 10% FBS. The assay plates were incubated in the presence/absence of Ig1&2-Fc or Fc control protein for 24 hours at 37°C and 5% CO_2_, after which the Transwell inserts were removed, and the upper chamber was gently wiped with a cotton swab to remove nonmigrating cells. Transwell membranes were fixed for 5 minutes in methanol and stained for 30 minutes in 0.2% crystal violet in 2% ethanol. Cells were imaged using a Keyence BZX710 microscope at 10× original magnification and quantified by counting 4 nonoverlapping fields using ImageJ software (NIH, version 1.8.0_201).

#### Invasion assays.

Cells for invasion assays were seeded as for migration assays into the upper chamber of a polyethylene terephthalate culture insert (pore size of 8 μm) precoated with ECM protein (354480; Corning). The assay plates were allowed to incubate and stained as described for migration assays. Cells were imaged using a Nikon Eclipse E800 at 4× original magnification and quantified by counting 2 nonoverlapping fields using ImageJ software.

### Scratch wound assays

A total of 5.0 × 10^4^ cells were seeded into a 12-well plate and allowed to adhere. Cells were starved overnight in DMEM containing 1% FBS, and on the following day, scratched with a 1 mL tip and incubated in DMEM containing 5% FBS in the presence of 100 nM Ig1&2-Fc or Fc control protein. Wells were imaged with a BioTek Cytation 5 Imaging Reader (Agilent) and analyzed using ImageJ software to count the number of cells migrating past the wound after 6 hours.

### Mice

BALB/c (BALB/cAnNTac) and NOD (NOD/MrkTac) were acquired from Taconic Biosciences. C57BL/6J (000664) were acquired from Jackson Laboratory.

### Mouse toxicology study

#### Serum assays.

Serum was collected via retro-orbital blood collection at the indicated time points of the study. ALT and AST activity levels were measured with ALT (MAK052) and AST (MAK055) activity assay kits (Sigma-Aldrich). Creatinine levels were measured with the Mouse Creatinine Assay Kit (80350; Crystal Chem). Sample concentrations were assigned a value of 0 mg/dL if the absorbance postreaction was lower than the initial absorbance. ANP levels were measured with the ANP ELISA Kit (EIA-ANP) from RayBiotech.

#### NCV tests.

At the beginning and end of the study, mice were anesthetized, and the sciatic nerve was surgically exposed via a 0.5 cm long incision in the flank followed by separation of underlying musculature by blunt dissection. A thermistor probe was placed adjacent to the nerve and the wound closed with a skin clamp. The nerve was stimulated (single 5v, 0.05 ms square wave pulse) by fine needle electrodes placed at the sciatic notch and Achilles tendon and the evoked electromyogram recorded from the interosseous muscles via 2 fine needle electrodes.

#### Tactile allodynia assessment.

Tactile allodynia was assessed using the Von Frey threshold method. Mice were placed on a mesh grid while Von Frey monofilaments of increasing buckling weight (0.16–6 g) were pushed steadily upon the plantar surface until the filament buckled. Movement of the paw in response to filaments of varying rigidity was recorded.

#### Thermal response.

Mice were placed in a glass-floored observation chamber with a movable controlled heat source that increases in temperature up to 30°C at a rate of ~1–1.5°C/s at the indicated time points. Hind paw withdrawal latency time in response to the heating stimulus was measured.

#### Corneal confocal microscopy.

Corneal nerve images were captured from each mouse at the indicated time points with the Heidelberg Retinal Tomograph with Rostock Corneal Module. Outlines of the imaged nerves were traced and total area quantified in FIJI software (NIH).

#### Cardiac assessment.

Electrocardiograms and echocardiograms were performed by the UCSD Seaweed Canyon Cardiovascular Physiology Laboratory.

### Mouse arthritis model

C57BL/6 KRN mice were provided by Christophe Benoist (Harvard Medical School, Boston, Massachusetts, USA) and were crossed with NOD mice to obtain arthritic offspring (K/BxN mice) whose sera were pooled for use in the K/BxN passive STIA (27). To elicit STIA, 8- to 10-week-old female BALB/c mice were injected retro-orbitally with 150 μL of arthritogenic K/BxN serum. Severity of arthritis was evaluated by clinical scoring (as described below) every other day, starting on the day of serum injection, unless otherwise stated. Arthritis was clinically scored in wrists and ankles as previously described (12): 0 = normal; 1 = minimal erythema and mild swelling; 2 = moderate erythema and mild swelling; 3 = marked erythema and severe swelling, digits not yet involved; and 4 = maximal erythema and swelling, digits involved. Proteins, vehicle control (Tris NaCl), or human IgG1-Fc control protein were administered according to the dose and schedule described in the figure legends.

### Histological scoring of mouse arthritic joints

Whole hind paws were fixed in 10% formalin, decalcified, trimmed, and embedded in paraffin. Paraffin-embedded samples were sectioned sagittally and trimmed to a depth in which the 4 joints in between tibia, talus, calcaneus, cuboid, and metatarsal were visible according to the SMASH recommendations (28). We prepared 4 μm–thick sections from this area, dried them overnight at 37°C, and baked at 60°C for 30 minutes to ensure adhesion of sections to the slides. Slides were stained with H&E using standard protocols (28). Histopathological scoring was performed as described (6, 12). Briefly, joints of arthritic mice were assigned scores of 0 to 4 for inflammation based on H&E staining, according to the following criteria: 0 = normal; 1 = minimal infiltration of inflammatory cells in periarticular area; 2 = mild infiltration; 3 = moderate infiltration; and 4 = marked infiltration. Joints of arthritic mice were given scores of 0 to 4 for bone resorption based on H&E staining, according to the following criteria: 0 = normal; 1 = minimal (small areas of resorption, not readily apparent on low magnification); 2 = mild (more numerous areas of resorption, not readily apparent on low magnification, in trabecular or cortical bone); 3 = moderate (obvious resorption of trabecular and cortical bone, without full-thickness defects in the cortex; loss of some trabeculae; lesions apparent on low magnification); and 4 = marked (full-thickness defects in the cortical bone and marked trabecular bone loss). Histologic analyses were performed in a blinded manner. Images of whole ankles were acquired using an Echo Revolve microscope, Zeiss Axioscan Z1, or Zeiss Axioscan 7 slide scanner and analyzed using Zen software (Zeiss).

### In vivo bioavailability assays

Eight-week-old female BALB/c mice were administered 0.75 mg of Ig1&2-Fc via retro-orbital injection. Blood was collected 1, 24, 72, and 168 hours postinjection via cardiac puncture, and serum was separated via centrifugation at 2,000*g*. Transparent, 96-well, half-area, high-binding plates (3690; Corning) were coated with 2.5 or 5 μg/mL (as stated in the figure legend) of S-Fab in Dulbecco’s PBS (DPBS; 21-031-CV; Thermo Fisher Scientific) overnight at 4°C. The following day, wells were blocked with 0.1% BSA in DPBS for 1 hour at room temperature. Samples were diluted 1:1,000 in blocking buffer, and standards were prepared at serial dilutions of 0.25–0.0078 μg/mL in 1:1,000 normal mouse serum in blocking buffer and allowed to incubate for 2 hours at room temperature. HRP-conjugated anti-human IgG Fcγ (32935S; CST) was added at a concentration of 1:20,000 in blocking buffer for 1 hour at room temperature. We added 3,3′,5,5′-Tetramethylbenzidine (TMB) substrate (7004P4; CST) for 20 minutes, followed by addition of STOP solution (7002P4; CST). Wells were washed with Tris-buffered saline with 1% Tween 20 at all relevant steps. Absorbance was read at 450 nM with a Tecan Infinite F Plex. Sample concentrations were assigned a value of 0 nM if the concentration was calculated to be less than 0 nM. To determine the half-life of each agent, the serum concentrations of 3 mice per time point were fit to a nonlinear 1-phase decay model in Prism software; the half-life was identified by the corresponding best-fit values.

### FLS IL-6 secretion assay

FLS were seeded in a 6-well plate until they reached 80% confluence. Cells were pretreated with 100 nM of the indicated proteins for 1 hour prior to stimulation with recombinant mouse TNF (410-MT; R&D Systems) for 24 hours. Supernatant was collected and spun down at 210*g* to remove debris. IL-6 secretion was measured according to the manufacturer’s instructions in the BioLegend ELISA Max Deluxe sets for mouse (431304; BioLegend) and human (430504; BioLegend) IL-6 using a Tecan Infinite F Plex. Samples were assigned a value of 0 if the relative concentration was calculated to be less than 0.

### Statistics

The unpaired 2-tailed *t* test, 1- or 2-way ANOVA, linear or nonlinear fit models, or Mann-Whitney *U* test was performed where appropriate as reported in the figure legends. All statistical analyses were performed using GraphPad Prism software. A comparison was considered significant if *P* was less than 0.05.

### Study approval

All animal experiments were carried out in accordance with the Institutional Animal Care and Use Committee–approved protocol at UCSD (S16098). RA FLS used in this study were previously obtained from discarded synovial tissue from patients with RA undergoing synovectomy, as approved by the UCSD Institutional Review Board under protocol 140175.

### Data availability

Values for all data points in graphs are included in the Supporting Data Values file.

## Author contributions

Conceptualization was done by SHR, ZZ, SMS, and NB. Methodology was done by SHR, ZZ, ES, SMS, and NB. Investigation was done by SHR, ZZ, MCL, THDR, ACS, MB, ND, KEF, MMH, JK, MZ, NAC, GSF, JWB, MNDS, ES, SMS, and NB. Resources were provided by NAC and GSF. Visualization was done by SHR, ZZ, MCL, THDR, MNDS, SMS, and NB. Funding acquisition was done by MNDS, SMS, and NB. Project administration was done by SHR, ZZ, ES, SMS, and NB. Supervision was done by NAC, MNDS, ES, SMS, and NB. Writing of the original draft was done by SHR, SMS, and NB. Review and editing were done by SHR, ZZ, MCL, THDR, ACS, MB, ND, KEF, MMH, JK, MZ, NAC, GSF, JWB, MNDS, ES, SMS, and NB. SHR and ZZ contributed equally to this research and are designated as co–first authors. SHR finalized this study and was identified to be listed first.

## Funding support

This work is the result of NIH funding, in whole or in part, and is subject to the NIH Public Access Policy. Through acceptance of this federal funding, the NIH has been given a right to make the work publicly available in PubMed Central.

- NIH (R01 AR066053 and P30 AR073761) to NB.
- Congressionally Directed Medical Research Programs (W81XWH1610751) to NB and (W81XWH2010733) to NB and SMS.
- Funds from the Sahlgrenska Academy (JNR2020), the Swedish Society for Medical Research (S19-0062), the King Gustaf V’s 80th Anniversary and IngaBritt och Arne Lundbergs Forskningsstiftelse Foundations awarded to MNDS.

## Supplementary Material

Supplemental data

Supporting data values

## Figures and Tables

**Table 1:**
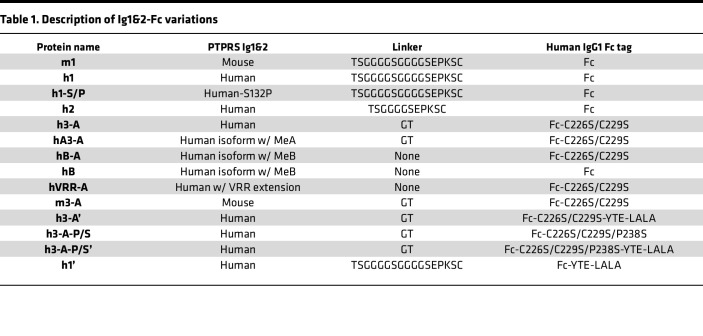
Description of Ig1&2-Fc variations

**Table 2:**
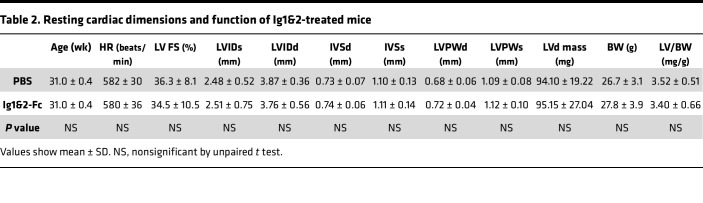
Resting cardiac dimensions and function of Ig1&2-treated mice

**Table 3:**
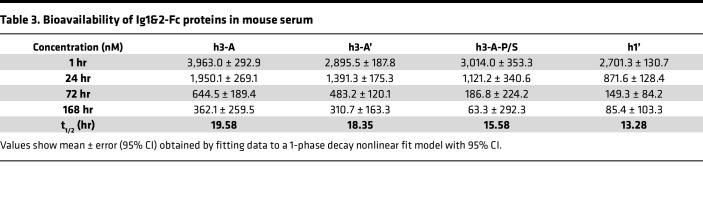
Bioavailability of Ig1&2-Fc proteins in mouse serum

**Table 4:**
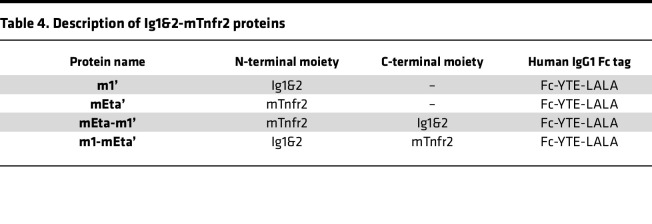
Description of Ig1&2-mTnfr2 proteins

## Version 1 09/30/2025

In-Press Preview

## Version 2 11/10/2025

Electronic publication

## References

[1] Alivernini S (2022). The pathogenesis of rheumatoid arthritis. Immunity.

[2] Greenberg JD (2012). A comparative effectiveness study of adalimumab, etanercept and infliximab in biologically naive and switched rheumatoid arthritis patients: results from the US CORRONA registry. Ann Rheum Dis.

[3] Jarvis B, Faulds D (1999). Etanercept: a review of its use in rheumatoid arthritis. Drugs.

[4] Feldmann M, Maini RN (2015). Perspectives from masters in rheumatology and autoimmunity: can we get closer to a cure for rheumatoid arthritis?. Arthritis Rheumatol.

[5] Lee DM (2007). Cadherin-11 in synovial lining formation and pathology in arthritis. Science.

[6] Doody KM (2015). Targeting phosphatase-dependent proteoglycan switch for rheumatoid arthritis therapy. Sci Transl Med.

[7] Stanford SM (2013). Protein tyrosine phosphatase expression profile of rheumatoid arthritis fibroblast-like synoviocytes: a novel role of SH2 domain-containing phosphatase 2 as a modulator of invasion and survival. Arthritis Rheum.

[8] Coles CH (2011). Proteoglycan-specific molecular switch for RPTPσ clustering and neuronal extension. Science.

[9] Shen Y (2009). PTPsigma is a receptor for chondroitin sulfate proteoglycan, an inhibitor of neural regeneration. Science.

[10] Aricescu AR (2002). Heparan sulfate proteoglycans are ligands for receptor protein tyrosine phosphatase sigma. Mol Cell Biol.

[11] Bunin A (2015). Protein tyrosine phosphatase PTPRS is an inhibitory receptor on human and murine plasmacytoid dendritic cells. Immunity.

[12] Svensson MND (2020). Synoviocyte-targeted therapy synergizes with TNF inhibition in arthritis reversal. Sci Adv.

[13] Davis PM (2007). Abatacept binds to the Fc receptor CD64 but does not mediate complement-dependent cytotoxicity or antibody-dependent cellular cytotoxicity. J Rheumatol.

[14] Pulido R (1995). The LAR/PTP delta/PTP sigma subfamily of transmembrane protein-tyrosine-phosphatases: multiple human LAR, PTP delta, and PTP sigma isoforms are expressed in a tissue-specific manner and associate with the LAR-interacting protein LIP.1. Proc Natl Acad Sci U S A.

[15] Dall’Acqua WF (2006). Properties of human IgG1s engineered for enhanced binding to the neonatal Fc receptor (FcRn). J Biol Chem.

[16] Liu R (2020). Fc-engineering for modulated effector functions-improving antibodies for cancer treatment. Antibodies (Basel).

[17] Dall’Acqua WF (2002). Increasing the affinity of a human IgG1 for the neonatal Fc receptor: biological consequences. J Immunol.

[18] Piguet PF (1992). Evolution of collagen arthritis in mice is arrested by treatment with anti-tumour necrosis factor (TNF) antibody or a recombinant soluble TNF receptor. Immunology.

[19] Williams RO (1992). Anti-tumor necrosis factor ameliorates joint disease in murine collagen-induced arthritis. Proc Natl Acad Sci U S A.

[20] Ji H (2002). Critical roles for interleukin 1 and tumor necrosis factor alpha in antibody-induced arthritis. J Exp Med.

[21] Ober RJ (2001). Differences in promiscuity for antibody-FcRn interactions across species: implications for therapeutic antibodies. Int Immunol.

[22] Ko S (2022). An Fc variant with two mutations confers prolonged serum half-life and enhanced effector functions on IgG antibodies. Exp Mol Med.

[23] Kang C (2018). A novel therapeutic anti-HBV antibody with increased binding to human FcRn improves in vivo PK in mice and monkeys. Protein Cell.

[24] Petkova SB (2006). Enhanced half-life of genetically engineered human IgG1 antibodies in a humanized FcRn mouse model: potential application in humorally mediated autoimmune disease. Int Immunol.

[25] Kabat EA Sequences of Proteins of Immunological Interest.

[26] Arnett FC (1988). The American Rheumatism Association 1987 revised criteria for the classification of rheumatoid arthritis. Arthritis Rheum.

[27] Monach PA (2008). The K/BxN arthritis model. Curr Protoc Immunol.

[28] Hayer S (2021). ‘SMASH’ recommendations for standardised microscopic arthritis scoring of histological sections from inflammatory arthritis animal models. Ann Rheum Dis.

