# Supplementary material for: Optimization of a synoviocyte-targeted biologic for inflammatory arthritis in combination or bispecific administration with TNF inhibitors
Source: JCI Insight. 2025 Sep 30;10(21):e192984. doi: 10.1172/jci.insight.192984 (PMC12643511; doi:10.1172/jci.insight.192984)
Supplement: Supplemental data [file jciinsight-10-192984-s121.pdf]

Supplementary Materials for

Optimization of synoviocyte-targeted biologic for inflammatory arthritis in combination or bispecific administration with TNF inhibitors

Sterling H. Ramsey, Zixuan Zhao, Megan C. Lee, Thales Hein da Rosa, Ava C. Schneider, Miriam Bollmann, Nour Dada, Katie Frizzi, May M. Han. Jaeyeon Kim, Martina Zoccheddu, Nigel A. Calcutt, Gary S. Firestein, James W. Bryson, Mattias N. D. Svensson, Eugenio Santelli, Stephanie M. Stanford*, and Nunzio Bottini*

*Address correspondence to nunzio.bottini@cshs.org and ststanford@health.ucsd.edu.

This PDF includes:

Supplemental Figure 1. Effect of varying linkers and species in inhibiting migration.

Supplemental Figure 2. Effect of Ig1&2-Fc in inhibiting scratch wound migration.

Supplemental Figure 3. Detection of Ig1&2-Fc by anti-PTPRS Fab.

Supplemental Figure 4. Therapeutic treatment of bispecific Ig1&2 fusion to mTnfr2.

Supplemental Table 1. Gross necropsy of mice from four-month toxicology study.

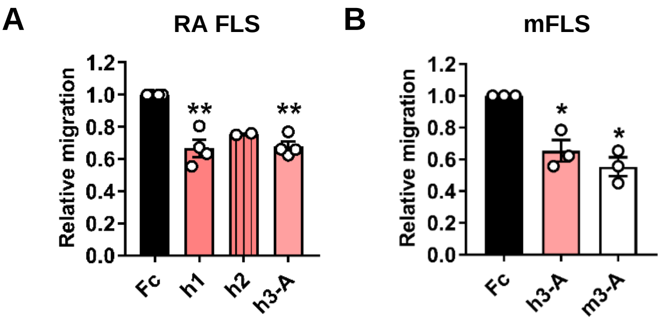

Supplemental Figure 1. Effect of varying linkers and species in inhibiting migration. (**A**) Relative transwell migration of RA FLS in response to 10% FBS in the presence of Ig1&2-Fc or Fc control protein (50 nM) (**h2**: *n* = 2; others: *n* = 4). (**B**) Relative transwell migration of mFLS in response to 10% FBS in the presence of Ig1&2-Fc or Fc control protein (100 nM) (*n* = 3). (**A** and **B**) Mean ± SEM relative to Fc control sample is shown. *$P < 0.05$ and **$P < 0.01$ by unpaired *t* test with Welch's correction (**A** and **B**).

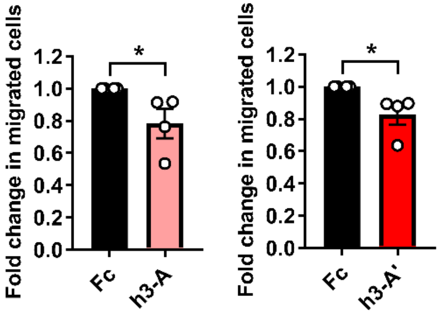

Supplemental Figure 2. Effect of Ig1&2-Fc in inhibiting scratch wound migration. Relative migration of RA FLS cells past scratch wound boundary in response to 5% FBS in the presence of Ig1&2-Fc or Fc control protein (100 nM) (*n* = 4). Mean ± SEM relative to Fc control sample is shown. *$P < 0.05$ by Mann-Whitney U test.

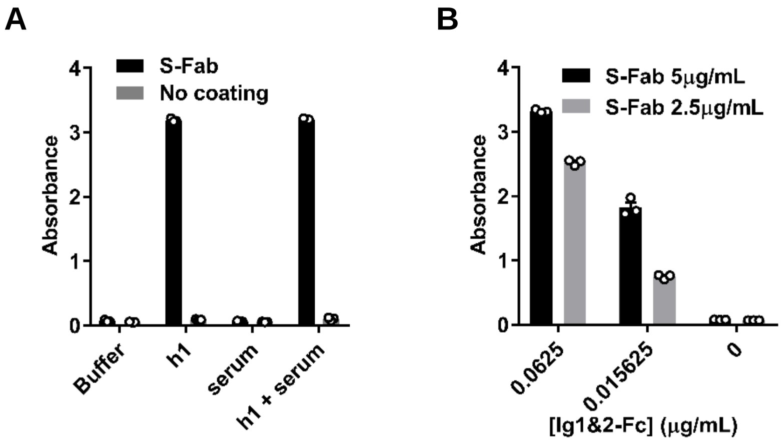

Supplemental Figure 3. Detection of Ig1&2-Fc by anti-PTPRS Fab. (**A**) Ig1&2-Fc protein in the presence or absence of mouse serum matrix was detected by coating with **S-Fab**. (**B**) Increased sensitivity of detection of Ig1&2-Fc was achieved by coating with a lower concentration of **S-Fab**. (**A** and **B**) Mean ± SEM of triplicate wells is shown.

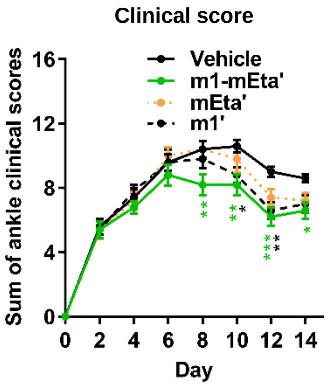

Supplemental Figure 4. Therapeutic treatment of bispecific Ig1&2 fusion to mTnfr2. Therapeutic treatment of BALB/c mice with K/BxN STIA by intraperitoneal injection of 0.3 mg (12 mg/kg) **m1-mEta'**, 0.1 mg (4 mg/kg) **mEta'**, 0.2 mg (8 mg/kg) **m1'**, or vehicle every 2 days for a total of 7 injections ($n = 5$). *$P < 0.05$, **$P < 0.01$, and ***$P < 0.001$ by two-way ANOVA with Dunnett's correction.

**Supplementary Table 1. Gross necropsy of mice from four-month toxicology study**		
**Description**	**(PBS)**	**Ig1&2-Fc (0.1 mg)**
**Skin**		
No obvious abnormalities	8/9	10/10
Rare focus of mast cells	1/9	- -
**Heart**		
No obvious abnormalities	4/9	4/10
Abnormal eosinophilic cardiomyocytes	4/9	4/10
Capillary congestion	1/9	2/10
**Lungs**		
Congestion*	2/9	1/10
Collapse*	4/9	4/10
Hemorrhage	5/9	5/10
Few foci of inflammation	2/9	3/10
Foci of inflammation	1/9	2/10
Few foci of pulmonary edema	- -	1/10
Adenoma	1/9	1/10
*Observed congestion and collapse may be due to lung fixation during processing		
**Liver**		
Rare foci of portal and parenchymal inflammation	3/9	3/10
Rare foci of portal or parenchymal inflammation with capillary congestion	- -	2/10
Mild portal inflammation with or without minimal foci of parenchymal inflammation	3/9	3/10
Bridging portal inflammation	1/9	- -
Very little inflammation	2/9	2/10
**Kidney**		
No obvious abnormalities	6/9	7/10
Focus of inflammation	2/9	1/10
Not seen in section	1/9	2/10
**Bladder**		
No obvious abnormalities	7/9	7/10
Not seen in section	2/9	3/10

**Spleen**		
Extra medullary hematopoiesis	9/9	10/10
**Thymus**		
No obvious abnormalities	9/9	10/10
**Salivary glands**		
No obvious abnormalities	9/9	10/10
**Stomach**		
No obvious abnormalities	8/9	10/10
Focus of inflammation	1/9	- -
**Pancreas**		
No obvious abnormalities	8/9	7/10
Abnormal foci in exocrine glands	- -	1/10
Rare foci of pale exocrine glands	- -	1/10
Rare abnormal foci in parenchyma	- -	1/10
Abnormal vacuoles in exocrine glands	1/9	- -
**Small intestine (duodenum & ileum)**		
No obvious abnormalities	6/9	7/10
Polyp	1/9	1/10
Focus of inflammation	1/9	- -
Polyp; inflammation	- -	1/10
Enlarged Peyer's patch	1/9	- -
Peyer's patch reactive with prominent macrophages	- -	1/10
**Prostate**		
No obvious abnormalities	3/4	3/5
Not seen in section	1/4	2/5
**Seminal vesicles**		
No obvious abnormalities	4/4	4/5
Inflammation	- -	1/5
Polyp, hyperplasia	- -	- -
**Ovary**		
Epithelial inflammation	3/5	3/5
Not seen in section	2/5	2/5
**Uterus**		

No obvious abnormalities	2/5	2/5
Dilated	- -	- -
Not seen in section	3/5	3/5
**Cervix**		
Epithelial inflammation	3/5	4/5
Not seen in section	2/5	1/5
